# Analysis of a Cochlear Implant Database: Changes in Tinnitus Prevalence and Distress After Cochlear Implantation

**DOI:** 10.1177/23312165221128431

**Published:** 2022-09-25

**Authors:** Kelly K. S. Assouly, Adriana L. Smit, Robert H. Eikelboom, Cathy Sucher, Marcus Atlas, Robert J. Stokroos, Inge Stegeman

**Affiliations:** 1 Department of Otorhinolaryngology and Head & Neck Surgery, University Medical Center Utrecht, Utrecht, The Netherlands; 2 UMC Utrecht Brain Center, University Medical Center Utrecht, Utrecht University, Utrecht, The Netherlands; 3 Cochlear Technology Centre, Mechelen, Belgium; 4 104182 Ear Science Institute Australia, Subiaco, Western Australia; 5 Centre for Ear Sciences, Medical School, The University of Western Australia, Nedlands, Western Australia; 6 Department of Speech Language Pathology and Audiology, University of Pretoria, Pretoria, South Africa; 7 Curtin Medical School, Curtin University, Bentley, Western Australia; 8 Department of Ophthalmology, University Medical Center Utrecht, Utrecht, The Netherlands; 9 Epidemiology and Data Science, Amsterdam University Medical Center, University of Amsterdam, Amsterdam, The Netherlands

**Keywords:** adults, cochlear implant, prevalence, quality of life, tinnitus

## Abstract

The aim of this study was to estimate the prevalence and distress of tinnitus pre- and post-cochlear implantation in patients with bilateral severe to profound hearing loss. In this retrospective study, we included patients from a cochlear implant clinic in Perth, Western Australia. Pre- and post-cochlear implantation data from 300 implant recipients were collected on self-reported presence of tinnitus, tinnitus distress using the Tinnitus Reaction Questionnaire (TRQ), hearing-related quality of life using the Abbreviated Profile of Hearing Aid Benefit (APHAB), and consonant-nucleus vowel-consonant (CNC) word recognition test scores. Retrospectively, patients were grouped into those with or without tinnitus, and the grade of tinnitus distress. The potential factors associated with post-implantation changes in the presence of tinnitus and its distress were evaluated. Tinnitus prevalence was 55.8% pre-operatively and 44.3% post-implantation with a median TRQ score respectively of 12.0 (IQR: 1.0–28.0) and 3.5 (IQR: 0.0–16.2) points. Among the 96 patients experiencing tinnitus pre-implantation, 14.6% patients experienced moderate to catastrophic tinnitus distress pre-implantation compared to 6.3% post-implantation. To conclude, the pre- and post-implantation median TRQ score for the cohort population showed that tinnitus was a “slight” handicap. Tinnitus prevalence and its associated tinnitus distress decreased post-implantation. Patients with tinnitus post-implantation were significantly younger and had less severe pre-implantation hearing loss in the non-implanted ear than patients without tinnitus. Further research is needed to understand the factors influencing changes in tinnitus.

## Introduction

Tinnitus is the perception of a sound in the ears or head without an external auditory input (Baguley et al., 2013). It has a prevalence ranging from 10 to 30% of the general population with up to 3% of people with tinnitus experiencing severe and bothersome tinnitus resulting in a substantial reduction of the quality of life (Baguley et al., 2013; McCormack et al., 2016; Stegeman et al., 2021). The cause and mechanisms of tinnitus are still not well understood. However, hearing loss has often been associated with tinnitus, and identified as a most common risk factor (Eggermont & Roberts, 2015; Nondahl et al., 2011). In a recent retrospective cohort study, it was found that around 20% of adult patients having an initial hearing consultation at a single tertiary hearing institute report tinnitus as a primary complaint (Lewis et al., 2020). Amongst cochlear implant (CI) candidates, tinnitus prevalence has been reported at levels up to 52% to 86% (Baguley & Atlas, 2007; Pierzycki et al., 2016; Quaranta et al., 2004).

The CI is a device that partially restores hearing for people with severe-to-profound hearing loss by electrical stimulation of the auditory nerve. While some studies show that tinnitus loudness, distress or annoyance can be reduced or suppressed after cochlear implantation, others report that tinnitus can also be worsened in up to 10% of recipients (Quaranta et al., 2004; Ramakers et al., 2015). Induction of tinnitus has been reported in up to 4% of patients receiving a CI for bilateral severe to profound hearing loss (Quaranta et al., 2004). To date, no randomized controlled trials investigating cochlear implantation and its effect on tinnitus as a primary complaint have been reported. In several systematic reviews, authors were unable to definitively comment upon the effect of cochlear implantation on tinnitus due to the high degree of heterogeneity in study designs and studied populations, limited sample sizes, short follow-up durations, and differences in CI types and outcomes measures (Assouly et al., 2021; Peter et al., 2019; Ramakers et al., 2015). As the effect of cochlear implantation on tinnitus distress seems to vary widely between studies, it is of clinical importance to understand the factors underlying this variability. The variability of tinnitus outcomes following cochlear implantation may be associated with patient characteristics, trauma provoked by the implantation procedure, and the presence of tinnitus and/or tinnitus distress prior to surgery (Dixon et al., 2020; Hoekstra et al., 2014; Kim et al., 2016). A few researchers have addressed this issue and attempted to find predictive factors for the effect of cochlear implantation on tinnitus perception amongst individuals with bilateral severe-to-profound hearing loss. Poorer pre-implantation hearing thresholds (Dixon et al., 2020), or speech perception (Ramakers et al., 2018) were identified as potential predictive factors for tinnitus improvement after cochlear implantation. Some pre-implantation patient characteristics have also been reported to predict clinically relevant tinnitus improvement or suppression after cochlear implantation: unilateral localization of tinnitus (Ramakers et al., 2018), higher pre-implantation tinnitus severity (Dixon et al., 2020; Kim et al., 2016) or a less severe depression state (Kim et al., 2016). A larger deterioration of residual hearing at 250 Hz, i.e., the difference in hearing threshold before and after surgery at this frequency, has also been associated with tinnitus suppression (presence of tinnitus pre-implantation and complete absence of tinnitus post-implantation) (Ramakers et al., 2018). In contrast, Kloostra et al. were not able to find predictors for a positive tinnitus outcome, using speech comprehension scores and pre-operative tinnitus distress, personality characteristics, anxiety and depression, and hearing handicap questionnaires, although they did find predictors that negatively influence tinnitus outcome in terms of lower pre-implantation tinnitus handicap and hearing handicap (Kloostra et al., 2018). None of the factors identified in the abovementioned studies were consistent among the various prediction models, which might be partly due to the small sample sizes of studies, high risk of bias of the presented models and lack of validation of these models. Therefore, no consensus has been reached on factors predictive of tinnitus outcome post-implantation.

Since there is uncertainty on tinnitus prevalence post-implantation and there is no clear prediction model for presence of tinnitus and associated distress, this topic must be further investigated. Identifying key factors which can characterize tinnitus changes after implantation will help clinicians to counsel CI candidates on the risk of developing or improving tinnitus after implantation and thus help to manage patient expectations. Therefore, the primary aim of the study was to estimate the prevalence and distress of tinnitus pre- and post-implantation in patients with bilateral severe to profound hearing loss. The secondary aim was to assess potential factors associated with post-implantation changes in the presence of tinnitus and its distress. Finally, we compared patient and hearing-related factors between patients with and without tinnitus.

## Methods

### Study Population

A retrospective, longitudinal study was conducted. For this purpose, we reviewed a dataset gathered from 300 adult CI recipients with bilateral severe to profound hearing loss who were surgically implanted unilaterally or bilaterally with a CI between 2000 and 2017 at the Ear Science Clinic, Perth, Western Australia. The dataset is the same as the one used for a report on the association between tinnitus after cochlear implantation and hearing-related quality of life (Opperman et al., 2020). This population consisted of patients with pre-lingual and post-lingual deafness; pre-lingual deafness was defined as hearing loss occurring prior to three years of age. Patients followed a rehabilitation and follow-up plan after implantation that included auditory evaluations and questionnaires. Only patients who replied to the question about the presence or absence of tinnitus pre-operatively were included in the study.

### Data Collection and Handling

This study used data gathered from the patient records including outcomes of standardized questionnaires. Data were extracted from electronic databases by an authorized member of the research team and anonymized prior to viewing and analyses by other members of the research team. Data were captured pre-implantation and at 6 and 12 months after implantation, and then annually. Due to missing data for recipients at some follow-up time points, the data from the latest available time point after implantation were used for the analysis as the post-implantation follow-up. We considered the first implantation date as the surgery date for all questionnaires and measurements follow-up. In case of bilaterally implanted recipients, the post-implantation follow-up used in the analysis for tinnitus outcome was always when bilaterally implanted recipients had received both implants. Six bilaterally implanted recipients reported tinnitus suppression before their second implantation and did not have post-second implant score available. Two bilaterally implanted recipients did not have post-second implant scores available. We considered the post-implantation outcomes of these eight bilaterally implanted recipients as missing data.

### Outcome Assessement

As part of the pre- and post-implantation assessments, patients were asked to answer two single questions: “Are you currently experiencing tinnitus or have you experienced tinnitus in the past month?” and “How often have you experienced tinnitus in the past month?”. If the answer to the first question was positive, and the answer to the second question indicated that tinnitus was experienced more than very occasionally, then the patient was included in the self-reporting tinnitus group and was asked to complete the TRQ. Otherwise, the patient was included in the no tinnitus group and was not asked to complete the TRQ.

### Description of Variables

#### Outcome Variables

The Tinnitus Reaction Questionnaire (TRQ) is a measure of the psychological distress associated with tinnitus. The TRQ contains 26 questions divided in four subscales: general distress, interference with daily activities, severity of tinnitus, and avoidance (Meric et al., 1997; Wilson et al., 1991). Possible answers are: not at all (scored 0), a little of the time (scored 1), some of the time (scored 2), a good deal of the time (scored 3), and almost all of the time (scored 4). A total score can range from 0 to 104 points which are classified into five grades of severity (Wilson et al., 1991): slight (0 to 16 points), mild (18 to 36 points), moderate (38 to 56 points), severe (58 and 76 points) and catastrophic (78 and 104 points). In addition to completion of the TRQ, patients were asked about the characteristics of their tinnitus: tinnitus side, regularity, awareness, and volume. Ipsilateral or contralateral tinnitus was determined based on comparison between the post-implant tinnitus side and the CI side. For bilaterally implanted recipients, we always considered it to be ipsilateral tinnitus. A patient was deemed to have a TRQ score of 0 at any of the pre-operative or post-operative points at which they self-reported the absence of tinnitus.

Hearing-related quality of life of CI recipients was assessed pre-implantation and at each post-implantation follow-up visit using the Abbreviated Profile of Hearing Aid Benefit (APHAB). The APHAB is a 24 item questionnaire comparing the difficulties of aided and unaided listening in everyday situations (Cox & Alexander, 1995). This questionnaire has been validated for hearing aid users (Cox & Alexander, 1995). The APHAB has often been used in CI recipients without being validated for the clinical population of CI recipients. The APHAB assesses the outcome in four domains: Ease of Communication (EC), Reverberation (RV), Background Noise (BN) and Aversiveness (AV). In the three first subscales (EC, RV and BN), speech communication in different environments are scored whereas the last subscale (AV) negative reactions to environmental sound are assessed. Seven answers are possible: always (99% of the time), almost always (87% of the time), usually (75% of the time), half the-time (50% of the time), sometimes (25% of the time), hardly ever (12% of the time) and never (1% of the time). An overall score as well as four sub-domain scores were obtained based on the addition of scores of negative descriptors and reversed scores for positive descriptors. The higher the score, the greater the perceived hearing disability and thus the lower the hearing-related quality of life (Cox & Alexander, 1995).

Speech recognition performance was evaluated using the consonant-nucleus-consonant (CNC) test (Peterson & Lehiste, 1962). The CNC test is a validated and common measure in the CI standard of care (Luxford, 2001). The patient was presented with a list of 25 words at 65 dBA in quiet, with the speaker 1 meter in front of the patient, at zero degrees azimuth, in a soundproof booth. Pre- and post-implantation tests were performed aided, with the device used by the patient at the time of the test. Responses were scored as the percentage of correct repeated words by the patient for each list. The test was performed pre-implantation and at each post-implantation follow-up visit.

Demographic information regarding sex, age at implantation, etiology of hearing impairment of the implanted ear, laterality of implantation and pre- or post-lingual onset of hearing loss were collected. Existence of balance concerns was assessed pre-implantation using a binary question. Clinical guidelines of the Ear Science clinic (Perth, Western Australia) are to consider bilateral implantation where medically and audiologically appropriate at 6 months post initial implant. Apart from questionnaires, audiometric data were retrieved from the patients’ medical files. The pre-implantation pure tone average (PTA) was calculated for each ear using the four-frequency average hearing loss (4FAHL, average of 0.5, 1, 2 and 4 kHz) in unaided condition, as well as the pre-implantation high frequency pure tone average (PTAHF) using the mean of the hearing thresholds at 4, 6, 8 kHz. Pure tone averages were classified by side of implantation (implanted or non-implanted ear). In case of bilateral implantation, the pure tone averages of both implanted ears were calculated.

#### Substantial TRQ Change Classification

We distinguished six categories of change in tinnitus status: no tinnitus reported (either pre- or post-implantation), total tinnitus suppression (tinnitus reported pre-implantation but not post-implantation), tinnitus induction (no tinnitus reported pre-implantation but reported post-implantation), tinnitus reduction, tinnitus worsening, and no tinnitus change.

Tinnitus reduction and tinnitus worsening are determined based on the difference in TRQ score pre- and post-implantation. A difference in TRQ score of 17 points between pre- and post-implantation, corresponding to a change of at least one severity grade on the TRQ score, was defined as a substantial TRQ change. A tinnitus worsening was characterized by an increase in TRQ score of more than 17 points post-implantation. Conversely, tinnitus reduction was considered when the patient reported a TRQ score of 17 points or more decrease than previous reports. No change is reported when the difference in TRQ score did not exceed 17 points.

### Statistical Analysis

Descriptive statistics were used to summarize patient characteristics in the tinnitus and no tinnitus groups. Normally distributed data were presented using mean and standard deviation (SD). Not normally distributed data were reported using median and interquartile range (IQR). APHAB, TRQ and CNC scores were considered as continuous outcome variables.

Wilcoxon signed rank tests were used to determine significant difference in TRQ scores between pre- and post-implantation time periods in the tinnitus group.

We used univariate linear regressions to assess the association between patient characteristics (tinnitus experience before implantation, age at implantation, sex, onset of deafness, balance concerns, lateralization of implantation, averaged hearing thresholds PTA and PTAHF in the implanted and non-implanted ear respectively) and the TRQ scores at 12 months post-implantation. The laterality of implantation was reported based on the situation of each recipient at 12 months after the first implantation.

Group differences pre- and post-implantation, were also evaluated between the tinnitus and no tinnitus groups in order to identify features that could statistically distinguish one group from another. Wilcoxon rank sum tests were used for continuous variables. Pearson chi square tests were used to assess the difference between categorical variables.

Statistical analysis between different tinnitus change groups were not performed because of the small sample size within each group. Missing data imputation was not used because we were not able to verify the nature of the missing data i.e., random or not.

All analyses were performed using R Studio version 1.3.1073 (®R Studio). A p-value lower than 0.05 indicated a statistically significant result. Corrections for multiple comparison correction were not performed.

## Results

### Cohort Characteristics

A total of 300 adults who underwent cochlear implantation between 2001 and 2016 were reviewed for the purpose of the study. The cohort characteristics are summarized in Table 1. The cohort APHAB and CNC outcomes are summarized in Supplemental Table S1. The median age was 65.0 years (IQR: 52.2−74.5), 52.3% (157/300) were men and 47.7% (143/300) were women. A high proportion of these were unilaterally implanted recipients (77.3%, 232/300). For these unilaterally implanted recipients, pre-implantation median PTA hearing thresholds were 92.5 dB HL (IQR: 80.9−103.8) and 77.5 dB HL (IQR: 60.0−90.6) in the implanted and non-implanted ear respectively. Overall, 75% (169/225) of CI users had post-lingual deafness and 33% (99/300) reported pre-implantation balance concerns. The mean time between the implantation date and the latest post-implantation follow-up was 468 days, i.e., 15.4 months, for unilaterally implanted recipients.

**Table 1.** Cohort Baseline Characteristics.

Characteristic	Cohort (n = 300, %)
Age at implantation, median (IQR)	65.0 (52.2−74.5)
Sex, n (%)	
Male	157 (52.3)
Female	143 (47.7)
Onset of hearing loss	
Pre-lingual, n (%)	56 (18.7)
Post-lingual, n (%)	169 (56.3)
Missing, n (%)	75 (25.0)
Balance concerns, n (%)	99 (33.0)
Etiology	
Congenital, n (%)	60 (20.0)
Hereditary, n (%)	60 (20.0)
Meniere's, n (%)	20 (6.7)
Noise exposure, n (%)	31 (10.3)
Otosclerosis, n (%)	20 (6.7)
Other, n (%)	70 (23.3)
Unknown, n (%)	16 (5.3)
Missing, n (%)	23 (7.7)
Laterality of implantation	
Unilateral	232 (77.3)
Bilateral	68 (22.7)
Pre-operative PTA in dB HL, median (IQR)	
Implanted ear (unilateral) (164)	92.5 (80.9 − 103.8)
Missing, n (%)	68 (29.3)
Non-implanted ear (unilateral) (215)	77.5 (60.0 − 90.6)
Missing, n (%)	17 (7.3)
Implanted ear (bilateral) (57)	
Left	101.2 (85.0 − 113.1)
Right	105.0 (86.2 − 117.5)
Missing, n (%)	11 (16.2)
Pre-operative PTAHF in dB HL, median (IQR)	
Implanted ear (unilateral) (111)	108.3 (96.7 − 115.0)
Missing, n (%)	121 (52.2)
Non-implanted ear (unilateral) (141)	96.7 (75.0 − 110.0)
Missing, n (%)	91 (39.2)
Implanted ear (bilateral) (38)	
Left	113.3 (108.3 − 116.7)
Right	113.3 (107.1 − 116.7)
Missing, n (%)	30 (44.1)

For the 68 bilaterally implanted recipients, the median interval between the two implantations was 746 days, i.e., 24.6 months, and the mean time between the second implantation date and the latest post-implantation follow-up was 590 days, i.e., 19.3 months. All bilaterally implanted recipients were implanted sequentially, except one who had been implanted simultaneously. Pre-implantation median PTA hearing thresholds were 101.2 dB HL (IQR: 85.0–113.1) and 105.0 dB HL (IQR: 86.2–117.5) in the left and right ears, respectively.

### Tinnitus Prevalence

Of the 300 patients, 172 (57.3%), 195 (65.0%), 124 (41.3%), 145 (40.3%), and 97 (32.3%) patients answered the single question about the presence of tinnitus at pre-implantation, 6, 12, 24, and 36 months post-implantation, respectively. Before implantation, 96 out of 172 (55.8%) patients reported tinnitus. The proportion of patients reporting tinnitus decreased over time (Table 2), with a prevalence decreasing from 55.8% pre-implantation to 44.3% 36 months post-implantation. Of the 96 patients who reported tinnitus pre-implantation, 27 (28.1%) did not report tinnitus at a later timepoint (Supplemental Figure S1). Of the 76 patients who did not report tinnitus prior to implantation, 14 (18.4%) reported tinnitus post-implantation (Supplemental Figure S1).

**Table 2.** Tinnitus Reported, TRQ Score and Tinnitus Characteristics Associated at Different Evaluation Time.

Variable	Pre-CI	6 months post-CI	12 months post-CI	24 months post-CI	36 months post-CI	Post-CI
N	172	195	124	145	97	280
Tinnitus, n (%)	96 (55.8)	98 (50.25)	61 (50.8)	67 (46.2)	43 (44.3)	124 (44.3)
No tinnitus, n (%)	76 (44.2)	97 (49.75)	63 (49.2)	78 (53.8)	54 (55.7)	156 (55.7)
Missing, n (%)	128 (42.7)	05 (35.0)	176 (58.7)	155 (51.7)	203 (67.7)	20 (6.7)
TRQ, median (IQR)	12.0 (1.0 − 28.0)	2.0 (0.0 − 12.0)	4.0 (1.0 − 13.8)	4.0 (0.0 − 11.0)	3.0 (0.0 − 9.0)	3.5 (0.0 − 16.3)
p‐value		<0.001^*^	<0.001^*^	<0.001^*^	0.14	<0.001^*^
Tinnitus side (unilateral CI), n (%)						
In both ears but worse in my left ear	6 (7.1)	11 (12.9)	8 (16.0)	6 (10.9)	5 (15.6)	14 (14.3)
In both ears but worse in my right ear	10 (11.9)	9 (10.6)	7 (14.0)	7 (12.7)	4 (12.5)	14 (14.3)
In both ears equally	12 (14.3)	15 (17.6)	8 (16.0)	12 (21.8)	9 (28.1)	19 (19.4)
In my head	11 (13.1)	13 (15.3)	6 (12.0)	7 (12.7)	3 (9.4)	17 (17.3)
Only in my left ear	20 (23.8)	11 (12.9)	4 (8.0)	12 (21.8)	6 (18.8)	14 (14.3)
Only in my right ear	14 (16.7)	19 (22.4)	12 (24.0)	8 (14.6)	5 (15.6)	15 (15.3)
Missing	11 (13.1)	7 (8.2)	5 (10.0)	3 (5.5)	0 (0.0)	5 (5.1)
Tinnitus side (bilateral CI), n (%)						
In both ears but worse in my left ear	1 (8.3)	1 (7.7)	0 (0.0)	1 (8.3)	0 (0.0)	3 (11.5)
In both ears but worse in my right ear	1 (8.3)	0 (0.0)	1 (9.1)	1 (8.3)	1 (9.1)	1 (3.8)
In both ears equally	5 (41.7)	5 (38.5)	1 (9.1)	4 (33.3)	4 (36.4)	6 (23.1)
In my head	3 (25.0)	0 (0.0)	6 (54.5)	3 (25.0)	4 (36.4)	9 (34.6)
Only in my left ear	0 (0.0)	0 (0.0)	1 (9.1)	1 (8.3)	2 (18.2)	4 (15.4)
Only in my right ear	0 (0.0)	3 (23.1)	0 (0.0)	1 (8.3)	0 (0.0)	2 (7.7)
Missing	2 (16.7)	4 (30.8)	2 (18.2)	1 (8.3)	0 (0.0)	1 (3.8)
Tinnitus regularity, n (%)						
Constant (is there all the time)	3 (55.2)	48 (49.0)	26 (42.6)	32 (47.8)	19 (44.2)	66 (53.2)
Intermittent (comes and goes)	43 (47.8)	50 (51.0)	35 (57.4)	35 (52.2)	24 (55.8)	58 (46.8)
Missing	0 (0.0)	0 (0.0)	0 (0.0)	0 (0.0)	0 (0.0)	0 (0.0)
Tinnitus volume, n (%)						
Changes in volume (goes softer and louder)	62 (64.6)	65 (66.3)	40 (65.6)	44 (65.7)	27 (62.8)	85 (68.5)
Stays at the same volume	34 (35.4)	33 (33.7)	21 (34.4)	23 (34.3)	16 (37.2)	39 (31.5)
Missing	0 (0.0)	0 (0.0)	0 (0.0)	0 (0.0)	0 (0.0)	0 (0.0)
Tinnitus awareness, n (%)						
All of the time	18 (18.8)	11 (11.2)	6 (9.8)	5 (7.5)	5 (11.6)	12 (9.7)
Most of the time	32 (33.3)	22 (22.4)	15 (25.6)	13 (19.4)	8 (18.6)	30 (24.2)
Some of the time	32 (33.3)	47 (48.0)	30 (48.2)	37 (55.2)	7 (16.3)	64 (51.6)
Hardly ever	14 (14.6)	18 (18.4)	10 (16.4)	12 (17.9)	23 (53.5)	18 (14.5)
Missing	0 (0.0)	0 (0.0)	0 (0.0)	0 (0.0)	0 (0.0	0 (0.0)

Post-CI corresponds to the latest available time point after implantation for every patient.

CI: cochlear implantation; IQR: interquartile range; n: number of patients; TRQ: Tinnitus Reaction Questionnaire.

N corresponds to the number of patients answering the question about tinnitus experienced. The p-value reported results from the Wilcoxon signed rank test between the TRQ score pre-implantation and the TRQ score post-implantation for every evaluation time.

* indicates variables that showed a significant difference with the TRQ score pre-implantation (p < 0.05)

### Tinnitus Characteristics

Of the 96 patients reported pre-implantation tinnitus, prior to implantation, 34 patients (35.4%) had unilateral tinnitus whilst 35 patients (36.4%) had bilateral tinnitus, and 14 (14.6%) reported central tinnitus (in the head). At the latest available time point post-implantation, 124 recipients were included in the self-reported tinnitus group, where 98 were unilaterally implanted and 26 were bilaterally implanted (Table 2). Of the 98 unilaterally implanted recipients, 29 had unilateral post-implantation tinnitus (25 ipsilateral tinnitus, 4 contralateral tinnitus), 64 had bilateral or central tinnitus (19 in both ears but worse in the ipsilateral ear, 9 in both ears but worse in the contralateral ear, 19 both ears equally and, 17 in the head), and 5 were unsure about the tinnitus location. Of the 26 bilaterally implanted recipients, 6 had unilateral tinnitus, 10 had bilateral tinnitus, 9 had central tinnitus, and 1 did not report his/her tinnitus location.

Post-implantation, variations in tinnitus volume, described as “goes softer and louder”, occurred in 85 patients (68.5%) whereas 39 patients (31.4%) reported a stable volume (Table 2). Sixty-six patients (53.2%) experienced constant tinnitus while the rest (46.8%) experienced tinnitus intermittently. Tinnitus awareness pre-implantation was reported as “all the time” by 18 (18.75%) of the participants, “most of the time” by 32 (33.3%), “some of the time” by 32 (33.3%) and “hardly ever” by 14 (14.6%). Post-implantation, 64 (51.6%) patients described their tinnitus awareness as “some of the time” and 12 (9.7%) described it as “all the time”.

### Tinnitus Distress

#### TRQ Score

A statistically significant reduction in TRQ score between pre-implantation and the latest available time point post-implantation was found (pre-implantation: 12.0 (IQR: 1.0−28.0); post-implantation: 3.5 (IQR: 0.0−16.3), Wilcoxon signed rank test, z = 1583, p <0.001) (Table 2, Supplemental Figure S2). Statistically significant changes in TRQ score were found at all individual post-implantation follow-up timepoints, except at 36 months post-implantation where the sample size was smaller (pre-implantation: 12.0 (IQR: 1.0−28.0); 6 months post-implantation: 2.0 (IQR: 0.0−12.0), Wilcoxon signed rank test, z = 973.5, p <0.001; 12 months post-implantation: 4.0 (IQR: 1.0−13.8), Wilcoxon signed rank test, z = 463, p < 0.001; 24 months post-implantation: 4.0 (IQR: 0.0−11.0), Wilcoxon signed rank test, z = 380, p < 0.001; 36 months post-implantation: 3.0 (IQR: 0.0−9.0), Wilcoxon signed rank test, z = 86.5, p = 0.14) (Table 2).

#### Tinnitus Severity Grade

**Figure 1.**
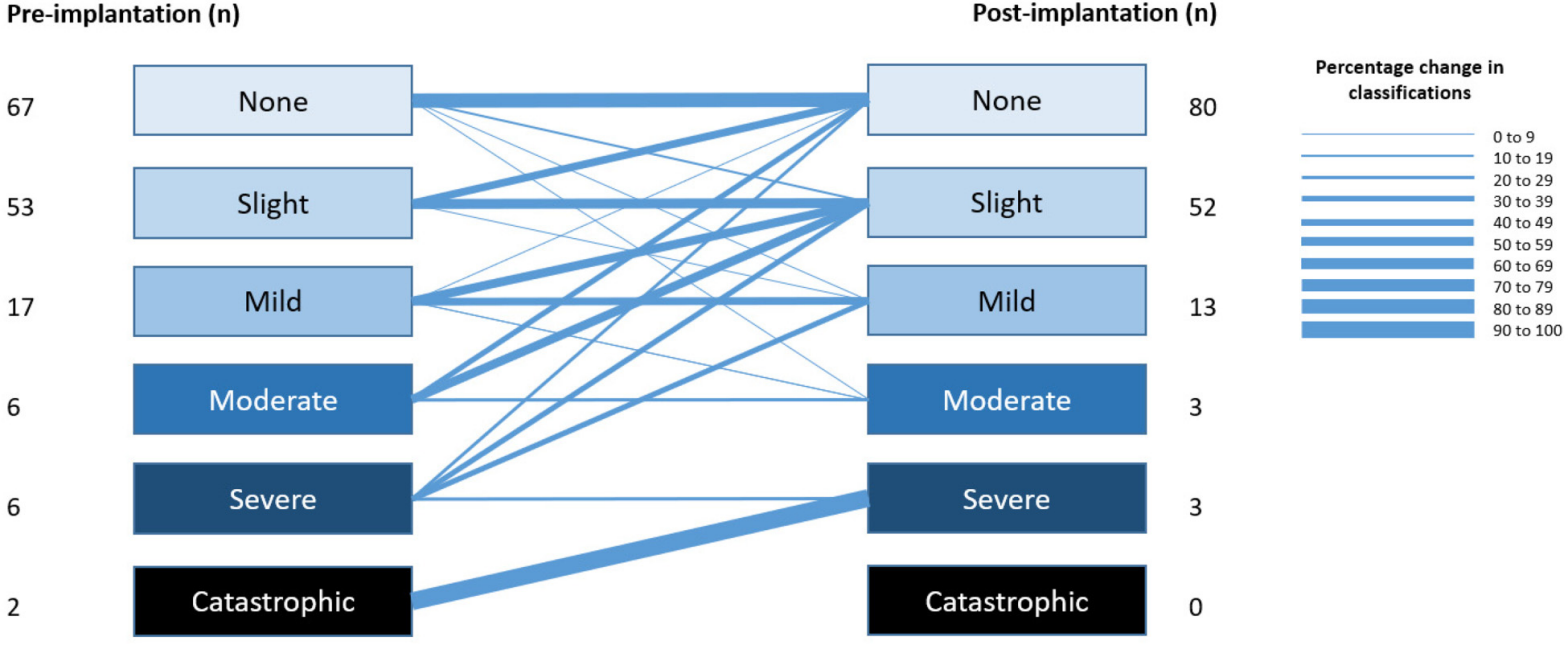
Pre- and post-implantation outcomes of the TRQ severity grades. TRQ severity grade classification: slight (0 to 16 points), mild (18 to 36 points), moderate (38 to 56 points), severe (58 to 76 points) and catastrophic (78 to 104 points).

The outcomes of the TRQ severity grades classification at the pre- and the latest available time point post-implantation are illustrated in Figure 1. Improvement in tinnitus severity grade was observed in 44 (28.9%) cases comparing pre-implantation versus post-implantation. Among the 6 patients with severe tinnitus prior to surgery, 5 (83.3%) reported a two grades reduction (severe to mild tinnitus). Sixteen (10.6%) patients scored a worsening of the tinnitus from none to a mild tinnitus grade.

#### Substantial TRQ Change

Pre- and post-implantation TRQ scores were available for a subset of 152 patients (Table 3). An examination of the substantial TRQ change showed that 27 (17.8%) had a total suppression of tinnitus (tinnitus reported pre-implantation but not post-implantation), 15 (9.9%) had a reduction of at least 17 points in TRQ score, 53 (34.9%) did not report tinnitus pre- or post-implantation, 14 (9.2%) had an induction of tinnitus, and 2 (1.3%) had a worsening of their tinnitus of at least 17 points in TRQ score compared to pre-implantation. The remaining 41 (27%) patients reported a change in TRQ score of less than 17 points, which was considered as no change (Table 3).

**Table 3.** Distribution of Characteristics and Scores Between Tinnitus Changes Groups.

Tinnitus changes	Induction	No change	No tinnitus	Reduction	Suppression	Worsening
Total, n (%)	14 (9.2)	41 (27.0)	53 (34.9)	15 (9.9)	27 (17.8)	2 (1.3)
Age (152), median (IQR)	65.8 (58.4 − 75.8)	68.5 (58.3 − 74.6)	71.9 (65.1 − 76.8)	58.2 (43.1 − 65.3)	62.5 (57.2 − 75.5)	45.3 (39.4 − 51.2)
Sex (152), n (%)						
Female	6 (42.9)	22 (53.7)	27 (50.9)	4 (26.7)	15 (55.6)	2 (100.0)
Male	8 (57.1)	19 (46.3)	26 (49.1)	11 (73.3)	12 (44.4)	
Laterality of implantation (152), n (%)						
Unilateral	12 (85.7)	33 (80.5)	44 (83.0)	15 (100.0)	25 (92.6)	2 (100.0)
Bilateral	2 (14.3	8 (19.5)	9 (17.0)	0 (0.0)	2 (7.4)	0 (0.0)
Balance concerns (152), n (%)						
No	12 (85.7)	29 (70.7)	40 (75.5)	7 (46.7)	20 (74.1)	2 (100.0)
Yes	2 (14.3)	12 (29.3)	13 (24.5)	8 (53.3)	7 (25.9)	
Onset of hearing loss (120), n (%)						
Post-lingual	7 (87.5)	29 (87.9)	35 (81.4)	10 (83.3)	16 (72.7)	
Pre-lingual	1 (12.5)	4 (12.1)	8 (18.6)	2 (16.7)	6 (27.3)	
Pre-operative PTA, median (IRQ)						
Implanted ear (107)	88.8 (78.1 − 98.8)	93.8 (82.8 − 104.1)	88.8 (80.6 − 105.6)	76.2 (70.0 − 96.2)	98.8 (89.1 − 105.0)	80.0 (68.1 − 91.9)
Non-implanted ear (121)	75.6 (56.9 − 85.9)	66.2 (38.8 − 85.0)	79.4 (65.3 − 91.2)	63.8 (15.0–80.0)	80.6 (63.4 − 95.3)	88.8 (88.8 − 88.8)
Pre-operative PTAHF, median (IRQ)						
Implanted ear (78)	103.3 (95.8 − 111.6)	98.3 (83.3 − 111.2)	109.4 (95.0 − 115.0)	90.0 (85.0 − 106.7)	111.7 (100.8 − 114.2)	106.7 (104.2 − 109.2)
Non-implanted ear (75)	96.7 (88.3 − 113.3)	81.7 (55.8 − 113.3)	103.3 (81.2 − 112.1)	75.0 (15.0 − 100.0)	91.7 (76.7 − 104.2)	103.3 (103.3 − 103.3)
Total ear (176), n (%)	16 (9.1)	49 (27.8)	63 (35.8)	15 (8.5)	31 (17.6)	2 (1.1)
CNC word, median (IQR)						
Pre-implantation (145)	10.0 (0.0 − 20.0)	0.0 (0.0 − 15.2)	8.0 (0.0 − 24.5)	4.0 (0.0 − 9.0)	4.0 (0.0 − 15.0)	24.5 (20.2 − 28.8)
6 months post (146)	32.0 (12.0 − 60.0)	40.0 (21.5 − 51.2)	25.0 (15.0 − 45.0)	27.0 (11.5 − 53.0)	37.5 (23.0 − 50.0)	17.5 (16.2 − 18.8)
12 months post (85)	35.0 (20.0 − 45.0)	40.0 (17.5 − 53.5)	30.0 (20.0 − 44.5)	24.5 (19.0 − 35.0)	34.0 (12.0 − 64.0)	12.0 (12.0 − 12.0)
Post − implantation (156)	35.0 (20.0 − 45.0)	40.0 (20.0 − 52.0)	32.0 (15.0 − 46.2)	24.5 (8.5 − 48.2)	42.5 (25 − 56.0)	7.5 (3.8 − 11.2)
APHAB, median (IQR)						
Pre − implantation (163)	72.0 (55.1 − 78.6)	55.2 (45.0 − 69.6)	60.7 (49.1 − 71.9)	65.8 (53.0 − 69.3)	54.1 (46.7 − 62.7)	49.2 (48.6 − 49.9)
6 months post (154)	45.3 (36.7 − 57.9)	36.0 (27.6 − 48.2)	40.8 (25.9 − 51.1)	49.2 (31.8 − 55.8)	38.0 (23.3 − 52.6)	53.3 (50.4 − 56.2)
12 months post (118)	44.1 (40.4 − 55.0)	33.2 (26.3 − 45.2)	34.5 (29.0 − 47.1)	41.6 (31.0 − 49.8)	36.4 (23.9 − 43.8)	45.3 (45.3 − 45.3)
Post − implantation (176)	39.4 (30.0 − 57.2)	35.5 (24.7 − 45.1)	36.9 (25.9 − 48.5)	42.6 (31.5 − 59.5)	34.9 (20.7 − 38.8)	46.4 (45.9 − 47.0)

The post-implantation scores correspond to the scores at the last available time point after implantation for every patient.

CI: cochlear implantation; IQR: interquartile range; n: number of patients; PTA: pure tone average; PTAHF: high frequency pure tone average.

#### Positive Substantial TRQ Changes

Patients experiencing tinnitus reduction or suppression after cochlear implantation demonstrated respectively a median pre-operative PTA of 76.2 dB and 98.8 dB and a median PTAHF of 90.0 dB and 111.7 dB in the implanted ear (Table 3). Patients experiencing positive substantial TRQ changes showed improvement in CNC word score post-implantation: tinnitus reduction group (baseline: 4.0 (IQR: 0.0–9.0); 12 months post-implantation: 24.5 (IQR: 19.0−35.0)) and tinnitus suppression group (baseline: 4.0 (IQR: 0.0−15.0); 12 months post-implantation: 34.0 (IQR: 12.0−64.0)). Improvement in APHAB scores post-implantation was observed for the tinnitus reduction group (baseline: 65.8 (IQR: 53.0−69.3); 12 months post-implantation: 41.6 (IQR: 31.0−49.8)) and the tinnitus suppression group (baseline: 54.1 (IQR: 46.7−62.7); 12 months post-implantation: 36.4 (23.9−43.8)) (Supplemental Table S3). For all APHAB subscales, an improvement was found post-implantation in the two groups with positive substantial TRQ changes (Table S3).

#### Negative Substantial TRQ Changes

Patients experiencing tinnitus worsening or induction after cochlear implantation demonstrated respectively a median pre-operative PTA of 80.0 dB and 88.8 dB and a median PTAHF of 106.7 dB and 103.3 dB in the implanted ear (Table 3). The two patients experiencing tinnitus worsening had bilateral tinnitus and worsening in CNC word score post-implantation (baseline: 24.5 (IQR: 20.2−28.8); 12 months post-implantation: 12.0 (IQR: 12.0−12.0)). The APHAB AV subscale score increased in patients reporting an induction or tinnitus worsening after implantation: tinnitus induction group (baseline: 26.1 (IQR: 16.4−41.7); 6 months post-implantation: 52.0 (IQR: 23.8−70.2); 12 months post-implantation: 47.8 (IQR: 40.2 − 56.2)) and tinnitus worsening group (baseline: 2.8 (IQR: 1.9−3.8); 6 months post-implantation: 14.6 (IQR: 11−17.5); 12 months post-implantation: 1.0 (1.0−1.0)) (Supplemental Table S3). In the two individuals with worsening tinnitus, the post-implantation APHAB RV scores were higher than the pre-implantation (baseline: 68.5 (IQR: 67.5−69.5); 6 months post-implantation: 81.8 (IQR: 77.1−86.4); 12 months post-implantation: 76.7 (IQR: 76.7−76.7)) (Supplemental Table S3). The APHAB EC and APHAB BN scores decreased over time for patients experiencing negative substantial TRQ change (Supplemental Table S3).

#### Associations Between Patient Characteristics and TRQ Score at 12 Months Post-Implantation

There was no significant association between the TRQ score 12 months post-implantation and other factors: tinnitus absence/presence pre-implantation, age at implantation, onset of hearing loss, pre-implantation balance concerns, laterality of implantation and pure tone averages in the implanted and the non-implanted ear (Table 4). More than 40% of subjects had missing data for the pure tone averages (24 (40.0%) for PTA and 34 (56.7%) for PTAHF in the implanted ear; 22 (40.0%) for PTAHF in the non-implanted ear).

**Table 4.** Relative Importance of Patient Characteristics on Tinnitus Distress at 12 Months Post-Implantation Measured Using Outcomes of Univariable Association Analysis.

Characteristic	Missing, n (%)	Cohort (n = 60)	OR (95% CI)	p-value
Tinnitus pre-implantation, n (%)	0 (0.0)		−1.63 (−12.86–9.59)	0.77
Yes		36 (60.0)		
No		24 (40.0)		
Age at implantation, median (IQR)	0 (0.0)	67.6 (57.1 − 74.5)	−0.09 ( − 0.38 − 0.19)	0.52
Sex, n (%)	0 (0.0)		−1.22 ( − 9.18 − 6.75)	0.76
Male		33 (55.0)		
Female		27 (45.0)		
Onset of hearing loss, n (%)	13 (21.7)		−3.01 ( − 15.27 − 9.26)	0.62
Pre-lingual		8 (13.3)		
Post-lingual		39 (65.0)		
Balance concerns pre-implantation, n (%)	0 (0.0)		1.40 ( − 6.91 − 9.71)	0.74
Yes		21 (35.0)		
No		39 (65.0)		
Laterality of implantation, n (%)	0 (0.0)		−2.67 ( − 17.01 − 11.67)	0.71
Unilateral		55 (91.7)		
Bilateral		5 (8.3)		
Pre-operative PTA in the implanted ear, median (IQR)	24 (40.0)	90.0 (80.0 − 98.8)	0.07 ( − 0.32 − 0.45)	0.73
Pre-operative PTA in the non-implanted ear, median (IQR)	4 (7.3)	72.5 (46.9 − 85.0)	0.05 ( − 0.10 − 0.20)	0.54
Pre-operative PTAHF in the implanted ear, median (IQR)	34 (56.7)	100.8 (91.2 − 110.0)	−0.33 ( − 0.76 − 0.10)	0.13
Pre-operative PTAHF in the non-implanted ear, median (IQR)	22 (40.0)	86.7 (65.0 − 111.7)	−0.02 ( − 0.17 − 0.13)	0.78

CI: confidence intervals; IQR: interquartile range; n: number of patients; PTA: pure tone average; PTAHF: high frequency pure tone average; TRQ: Tinnitus Reaction Questionnaire. The p-value reported results from the univariate linear regression modeling the TRQ score at 12 months post-implantation.

### Difference Between Tinnitus and no Tinnitus Group

#### Pre-Implantation

Patients with tinnitus pre-implantation were statistically significantly younger than patients without tinnitus (tinnitus group: 62.6 years (IQR: 45.3−74.3); no tinnitus group: 70.7 years (IQR: 59.7−76.7); Wilcoxon rank sum test, w = 4493, p = 0.009). Patients with tinnitus pre-implantation had statistically significantly less severe high-frequency hearing loss in the non-implanted ear (tinnitus group: 84.2 dB PTA (IQR: 61.7−110.0); no tinnitus group: 101.7 dB PTA (IQR: 85.0−111.7); Wilcoxon rank sum test, w = 1154.5, p = 0.03). There were no other statistically significant differences in all other patient characteristics between patients with and without tinnitus pre-implantation (Table 5).

**Table 5.** Distribution of Characteristics Between Tinnitus and no Tinnitus Reported pre- and Post-CI.

	Pre-CI			Post-CI		
Characteristic	No tinnitus 76 (44.2%)	Tinnitus 96 (55.8%)	p-value	No tinnitus 156 (55.7%)	Tinnitus 124 (44.3%)	p-value
Age, median (IQR)	70.7 (59.7 − 76.7)	62.6 (45.3 − 74.3)	0.009*	68.2 (57.3 − 76.2)	61.3 (47.7 − 72.0)	0.002*
Sex, n (%)			0.85			0.18
Female	40 (52.6)	48 (50.0)		79 (50.6)	52 (41.9)	
Male	36 (47.4)	48 (50.0)		77 (49.4)	72 (58.1)	
Laterality of implantation, n (%)			0.41			0.37
Unilateral	64 (84.2)	86 (89.6)		115 (73.7)	98 (79.0)	
Bilateral	12 (15.8)	10 (10.4)		41 (26.3)	26 (21.0)	
Balance concerns, n (%)			0.24			0.31
No	60 (78.9)	67 (69.8)		107 (68.6)	77 (62.1)	
Yes	16 (21.1)	29 (30.2)		49 (31.4)	47 (37.9)	
Onset of hearing loss, n (%)			0.77			0.20
Post-lingual	44 (80.0)	58 (76.3)		86 (72.9)	78 (81.2)	
Pre-lingual	11 (20.0)	18 (23.7)		32 (27.1)	18 (18.8)	
Pre-operative PTA, median (IQR)						
Implanted ear	92.5 (80.0 − 108.8)	93.8 (80.0 − 103.8)	0.96	95.0 (84.4 − 107.5)	93.8 (78.8 − 103.8)	0.09
Non-implanted ear	80.0 (62.5 − 91.2)	73.8 (43.8 − 90.0)	0.15	80.6 (68.4 − 95.0)	70.0 (50.0 − 85.0)	0.001*
Pre-operative PTAHF, median (IQR)						
Implanted ear	106.7 (95.0 − 114.7)	105.0 (91.2 − 113.2)	0.30	108.3 (96.7 − 115.0)	102.5 (85.0 − 112.5)	0.02*
Non-implanted ear	101.7 (85.0 − 111.7)	84.2 (61.7 − 110.0)	0.03*	103.3 (83.3 − 110.0)	85.8 (63.3 − 109.6)	0.01*

CI: cochlear implantation; IQR: interquartile range; n: number of patients; PTA: pure tone average; PTAHF: high frequency pure tone average.

The p-value reported results from statistical comparison test between the no tinnitus group and the tinnitus group. The Wilcoxon rank sum test was used for continuous variables and Person chi square test was used for categorical variables.

* indicates variables that showed a significant difference between the groups (p < 0.05)

#### Post-Implantation

Patients with tinnitus post-implantation were statistically significantly younger than patients without tinnitus (tinnitus group: 61.3 years (IQR: 47.7−72.0); no tinnitus group: 68.2 years (IQR: 57.3−76.2); Wilcoxon rank sum test, w = 11657, p = 0.002) (Table 5). Sex, laterality of implantation, balance concerns and onset of hearing loss did not differ significantly between groups. The non-tinnitus group had a statistically significantly more severe pre-implantation hearing loss in the non-implanted ear (tinnitus group: 70.0 dB PTA (IQR: 50.0−85.0); no tinnitus group: 80.6 dB PTA (IQR: 68.4−95.0); Wilcoxon rank sum test, w = 6138.5, p < 0.001). The same observation can be found in the high frequencies for both ears: implanted ear (tinnitus group: 102.5 dB PTA (IQR: 85.0−112.5); no tinnitus group: 108.3 dB PTA (IQR: 96.7−115.0); Wilcoxon rank sum test, w = 3948.5, p = 0.02), non-implanted ear (tinnitus group: 85.8 dB PTA (IQR: 63.3−109.6); no tinnitus group: 103.3 dB PTA (IQR: 83.3−110.0); Wilcoxon rank sum test, w = 2626.5, p = 0.01).

The APHAB and CNC word scores were not statistically significant discriminant factors between groups (Supplemental Table S2).

## Discussion

In this retrospective cohort study, we gathered data to estimate the prevalence and distress of tinnitus pre- and post-implantation among 300 patients with bilateral severe to profound hearing loss. Two hundred thirty-two (77.3%) patients underwent unilateral cochlear implantation, and 68 (22.7%) patients underwent bilateral cochlear implantation. Tinnitus prevalence was 55.8% preoperatively and 44.3% post-implantation. The median TRQ score was 12.0 (IQR: 1.0−28.0) points pre-implantation and 3.5 (IQR: 0.0−16.2) points post-implantation. Among the 96 patients experiencing tinnitus pre-implantation, 14.6% patients experienced moderate to catastrophic tinnitus distress pre-implantation. Post-implantation, 6.3% patients reported moderate to severe tinnitus distress. Patients with tinnitus post-implantation were statistically significantly younger and had less severe pre-implantation hearing loss in the non-implanted ear than patients without tinnitus.

About half of the CI patients (55.8%) experienced tinnitus pre-implantation in our cohort study. This finding suggests that tinnitus is more prevalent in CI candidates than in the general population (up to 30%) (McCormack et al., 2016). The estimate of the present study is in line with the prevalence of 52% reported in a sample of 211 UK adults identified as potential candidates for cochlear implantation (Pierzycki et al., 2016). Quaranta et al. reviewed studies on tinnitus experiences in patients undergoing cochlear implantation, which reported between 66% and 86% of CI recipients experiencing tinnitus before implantation (Quaranta et al., 2004). However, studies included in this review presented some considerable risks of bias including methodological limitations and heterogeneous populations. Post-implantation, we found an estimate of 44.3% CI users reported experiencing tinnitus. This is marginally lower than the 50% of tinnitus estimated in a UK Biobank resource (Pierzycki et al., 2016). One possible explanation for these discrepancies in tinnitus estimation is the differences in the study setting or the use of different definitions of tinnitus when assessing the presence of tinnitus (De Ridder et al., 2021). The scale of the “problem” of tinnitus in CI patients should not be defined by prevalence alone. Most patients in our cohort (80/151) experienced no distress post-implantation and only 6 out 151 patients experienced moderate to severe handicap. However, our finding may motivate stakeholders in the implementation of tinnitus counseling as part of the CI standard of care.

As described above, in the studied population of CI recipients, the experienced tinnitus distress was generally low. The median post-implantation TRQ score for our study population was 3.5, interpreted as no to slight handicap. Using the TRQ severity grade classification, 6.3% had moderate to severe tinnitus distress. Andersson et al. investigated the tinnitus handicap in 111 CI recipients with tinnitus, in which 24.5% experienced a moderate to severe handicap based on the classification of Tinnitus Handicap Inventory (Andersson et al., 2009). Among CI recipients, there might be a subgroup of users experiencing tinnitus as a problem after implantation. As such, attention should be paid to further characterize this group which could benefit from tinnitus specific therapy.

Comparing differences in patient characteristics between patients with post-implantation tinnitus and without post-implantation tinnitus revealed that patients with tinnitus were statistically significantly younger at implantation than patients without tinnitus. Previous studies did not find age at implantation as a discriminant factor (Dixon et al., 2020; Kim et al., 2016; Kloostra et al., 2018; Ramakers et al., 2018). Patients reporting tinnitus pre-implantation were also younger than patients without tinnitus. As most of patients reporting tinnitus pre-implantation were also in the group of patients reporting tinnitus post-implantation, this observation might be specific to the study sample. Thereby, this finding could be related to hearing levels. Baseline pure tone average was found as a discriminant factor between patients with tinnitus and without tinnitus pre- and post-implantation. The tinnitus group had better baseline hearing on average than no tinnitus groups. Within the tinnitus groups, patients experiencing a tinnitus reduction had better hearing thresholds pre-implantation. This outcome is not in agreement with the observation of Kompis et al. who reported that patients who develop tinnitus post-operatively had slightly better preoperative hearing thresholds in the implanted ear (Kompis et al., 2012). Further research with higher quality data is needed to assess whether pre-operative hearing loss could be meaningful for effect on tinnitus, especially at high frequencies. Speech perception, measured by CNC word score, was not significantly different between patients with tinnitus and without tinnitus. This observation is consistent with previous research on unilateral cochlear implantation (Amoodi et al., 2011).

No association was found between the TRQ score 12 months post-implantation and patient characteristics. Previous models predicting the effect of cochlear implantation on tinnitus distress assessed similar patient characteristics and did find significant associations. Dixon et al. (n = 358) showed that pure tone thresholds per 10-dB increase at 1 kHz (OR: 1.11 (95% CI: 1.00, 1.22)) and at 2 kHz (OR: 1.11 (95% CI: 1.01, 1.23)) in the contralateral ear were significantly associated with tinnitus improvement, defined as a reduction of at least 7 points in the Tinnitus Handicap Questionnaire, in unilateral CI users (Dixon et al., 2020). Further research is needed to identify key factors modeling the positive and negative effects of cochlear implantation on tinnitus and to direct clinical decision making and patient counselling, especially on the risk of tinnitus onset after implantation.

The prevalence of negative effects of cochlear implantation on experienced tinnitus, based on worsening of 17 points in TRQ score and induced tinnitus, was 10.5% in our study. These proportions are in agreement with previous studies, reporting any worsening in tinnitus distress scores in 4 to 13.7% (Kloostra et al., 2018; Kompis et al., 2012; Pan et al., 2009; Quaranta et al., 2004). The impact of tinnitus on cochlear implant performance and quality of life after implantation, as well as the risk of implantation inducing or worsening tinnitus is not well understood.

A novel finding of the present study was the absence of tinnitus severity grade worsening in patients with moderate or more severe tinnitus pre-implantation (Figure 1). This finding is in agreement with the association found between higher pre-implantation tinnitus burden, assessed by the Tinnitus Handicap Inventory, and tinnitus improvement in two studies attempting to predict the positive effect of cochlear implantation on tinnitus (Dixon et al., 2020; Kim et al., 2016). This observation suggests that tinnitus burden or distress should be an important criterion to consider when counselling about tinnitus worsening as a complication of cochlear implantation.

Our study has a relatively large sample size when compared with previous studies on tinnitus changes following cochlear implantation (Dixon et al., 2020; Kim et al., 2016; Kloostra et al., 2018; Kompis et al., 2012; Quaranta et al., 2004; Ramakers et al., 2018). The data were systematically collected at defined follow-up time points according to a strict process of data collection integrated in the standard of care of the clinic. We evaluated substantial tinnitus change based on a minimum difference in TRQ scores of 17 points (equivalent to a change in severity grade). This method enabled us to investigate substantial positive and negative effect on tinnitus and to classify tinnitus changes in five different categories: tinnitus suppression, tinnitus reduction, no tinnitus change, tinnitus worsening and tinnitus induction.

The most important limitation of this study is the lack of a pre-defined protocol, and the retrospective nature of this study. Data were collected in clinical care. This is also reflected in the high proportion of missing post-operative data at late follow-up time point (missing self-reported tinnitus: 58.7% (12 months post-CI); 51.7% (24 months post-CI); 67.7% (36 months post-CI)). We therefore choose to select data available of the latest follow-up point as the post-implantation data to compare it with the pre-implantation data collected.

In the present study, we assessed tinnitus based on two complementary variables: self-reported presence of tinnitus and TRQ score. This combination of outcomes highlights a limitation in the interpretation of the TRQ score. In fact, we encountered cases where patients reported they were experiencing tinnitus but had a TRQ score of 0 i.e., they reported no distress from their tinnitus. These cases would have been difficult to interpret based only on the TRQ questionnaire score. Furthermore, the TRQ questionnaire is a measure focusing on psychological distress associated with tinnitus and does not assess a broader construct of the impact of tinnitus or treatment-related changes, as could be measured using the Tinnitus Handicap Inventory (THI) or the Tinnitus Functional Index (TFI) (Boecking et al., 2021; Jacquemin et al., 2019).

Another limitation in our analysis was the interpretation of the difference in TRQ score. Previous studies have used the criteria of an improvement in TRQ score of 40% or greater as a clinically relevant tinnitus change (Hazell, 1999; McKinney et al., 1999). We think this criterion is meaningful for clinically relevant improvement but there is a missing criterion for clinically relevant increase in tinnitus distress. For this reason, we defined a new criteria equivalent to a change in tinnitus distress instead of a change in percentages. For our classification of tinnitus change categories, we used a difference in TRQ score of 17 points between two times of assessment, which corresponds to at least a change in tinnitus severity grade, as a substantial TRQ change. Conversely, no change is reported when the change in TRQ score does not exceed 17 points. The criterion must be validated before being extended to further studies using the TRQ as a measure of treatment-related change. Furthermore, since significant tinnitus worsening was not specifically defined in literature, tinnitus worsening is usually not considered during tinnitus questionnaire development. There is a need to develop research quantifying tinnitus worsening, which is an essential aspect in tinnitus treatment-related change.

Our study confirms the high prevalence of tinnitus in CI candidates and current CI recipients. Most CI recipients experienced no to slight tinnitus distress. The post-implantation median tinnitus distress was 3.5 on a TRQ scale of 100, which is in line with earlier studies in similar patient groups (Andersson et al., 2009; Ramakers et al., 2017, 2015). However, there is a subgroup of CI recipients experiencing tinnitus burden. Identifying these patients and addressing their needs should be a priority to ensure the benefit of cochlear implantation. Among the studied outcomes, no factor was associated with post-implantation tinnitus changes. Fully understanding tinnitus worsening and induction after cochlear implantation requires further research, which is essential to allow clinicians to be confident in clinical decision making and provide realistic expectations on tinnitus changes after implantation.

Multi-center studies with a larger data set may provide further information about tinnitus in patients with CIs. These may give insights in the importance of patient characteristics on tinnitus, its distress, and the possibilities to minimize negative outcomes after implantation. Perhaps more importantly, better quality data is required i.e., fewer missing data, agreement on definitions, standard tools to assess and grade tinnitus. To avoid selection bias, prospective data collection should aim not only to assess hearing performance in CI recipients but also to collect tinnitus information as a standard in implant clinics.

## Conclusion

Tinnitus prevalence was 55.8% preoperatively and 44.3% post-implantation. The median TRQ score was 12.0 (IQR: 1.0–28.0) points pre-implantation and 3.5 (IQR: 0.0–16.2) points post-implantation, interpreted as a “slight” tinnitus distress (TRQ < 17 points). A small proportion of recipients (6.3%) experienced tinnitus as moderate to severe post-implantation. Although, tinnitus distress in those reporting tinnitus pre-implantation improved statistically significantly post-implantation, there is no association between speech performance, measured by CNC word, and tinnitus distress, measured by TRQ. None of the patients reporting moderate to catastrophic tinnitus distress prior to implantation experienced worsening of tinnitus after implantation. The need to conduct research to fully understand tinnitus worsening and induction after cochlear implantation is important to extend our knowledge in order to allow clinicians to be confident in clinical decision making and provide realistic expectations on tinnitus changes after implantation. There is a need to combine the experiences of patients and clinical specialists involved in tinnitus management with evidence from around the world to better understand the impact of tinnitus on CI users.

## Supplemental Material

sj-docx-1-tia-10.1177_23312165221128431 - Supplemental material for Analysis of a Cochlear Implant Database: Changes in Tinnitus Prevalence and Distress After Cochlear Implantation Click here for additional data file. Supplemental material, sj-docx-1-tia-10.1177_23312165221128431 for Analysis of a Cochlear Implant Database: Changes in Tinnitus Prevalence and Distress After Cochlear Implantation by Kelly K. S. Assouly, Adriana L. Smit and Robert H. Eikelboom, Cathy Sucher, Marcus Atlas, Robert J. Stokroos, Inge Stegeman in Trends in Hearing
