# Supplementary material for: Analysis of a Cochlear Implant Database: Changes in Tinnitus Prevalence and Distress After Cochlear Implantation
Source: Trends Hear. 2022 Sep 25;26:23312165221128431. doi: 10.1177/23312165221128431 (PMC9515522; doi:10.1177/23312165221128431)
Supplement: sj-docx-1-tia-10.1177_23312165221128431 - Supplemental material for Analysis of a Cochlear Implant Database: Changes in Tinnitus Prevalence and Distress After Cochlear Implantation [file sj-docx-1-tia-10.1177_23312165221128431.docx]

# **Supplemental material**


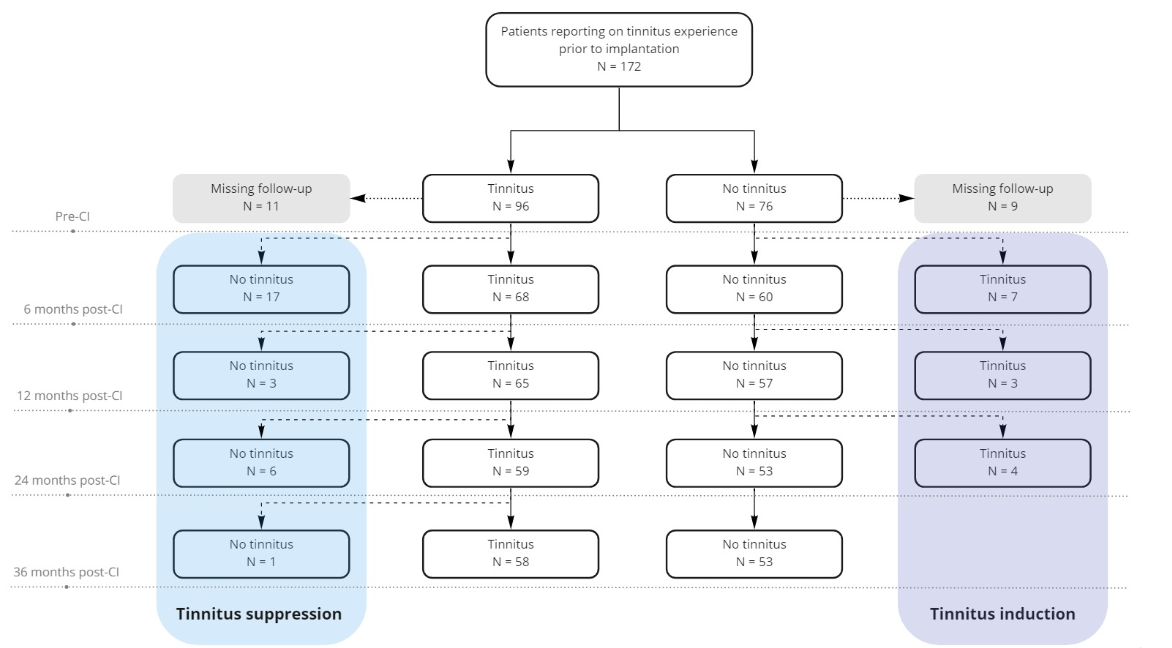


**Figure S1**. Distribution of tinnitus reporting pre-implantation and at 6, 12, 24 and 36 months post-implantation. CI: cochlear implantation; N: number of patients.


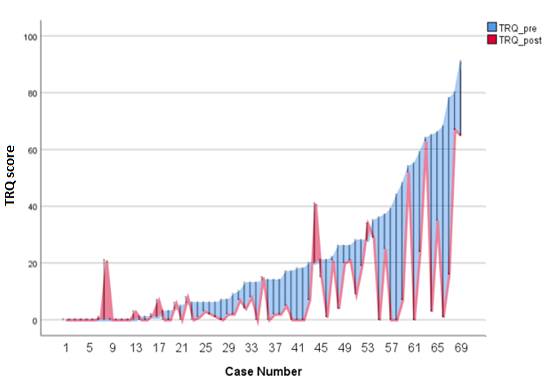


**Figure S2**. Pre- and post-implantation TRQ score of 68 patients who reported tinnitus pre- and post-implantation. Case number corresponds to individual ranged from 1 to 68.

The area in blue corresponds to a decrease in TRQ scores between pre- and post-implantation and the area in red corresponds to an increase in TRQ score between pre- and post-implantation.

| Outcomes | Ears (n = 567) |  |
| --- | --- | --- |
| Abbreviated Profile of Hearing Aid Benefit (APHAB), median (IQR)  Pre-implantation  Missing, n (%) | 60.6 (48.2-70.4)  178 (31.4) |  |
| 6 months post-implantation  Missing, n (%) | 38.2 (25.9-51.5)  279 (49.2) |  |
| 12 months post-implantation  Missing, n (%) | 35.6 (26.0-49.3)  355 (62.6) |  |
| 24 months post-implantation  Missing, n (%) | 35.4 (24.1-48.2)  305 (53.8) |  |
| 36 months post-implantation  Missing, n (%) | 36.8 (24.9-49.4)  395 (69.7) |  |
| CNC word score, median (IQR)  Pre-implantation  Missing, n (%) | 4.0 (0.0-16.0)  167 (29.5) |  |
| 6 months post-implantation  Missing, n (%) | 36.0 (20.0-53.5)  171 (30.2) |  |
| 12 months post-implantation  Missing, n (%) | 40.0 (25.0-60.0)  289 (51.0) |  |
| 24 months post-implantation  Missing, n (%) | 45.0 (32.0-60.0)  261 (46.0) |  |
| 36 months post-implantation  Missing, n (%) | 48.0 (35.0-60.0)  374 (66.0) |  |

**Table S1**. Cohort APHAB and CNC outcomes pre-implantation and at 6, 12, 24 and 36 months post-implantation.

| Characteristic | | No tinnitus post-CI | Tinnitus post-CI | p-value |
| --- | --- | --- | --- | --- |
| Total ears (355), n (%) | | 203 (57.2) | 152 (42.8) |  |
| CNC word, median (IQR) | |  |  |  |
| Pre-implantation (274) | | 5.0 (0.0 to 15.0) | 4.0 (0.0 to 16.0) | 0.56 |
| 6 months post (280) | | 35.0 (16.0 to 50.5) | 32.5 (20.0 to 52.8) | 0.79 |
| 12 months post (191) | | 40.0 (24.0 to 60.0) | 35.0 (21.0 to 55.0) | 0.39 |
| Post-implantation (318) | | 42.0 (25.0 to 60.0) | 40.0 (23.0 to 52.8) | 0.17 |
| APHAB, median (IQR) | |  |  |  |
| Pre-implantation (255) | | 57.4 (47.7 to 67.6) | 62.7 (47.4 to 73.5) | 0.17 |
| 6 months post (240) | | 38.5 (24.2 to 50.9) | 39.8 (29.8 to 53.3) | 0.14 |
| 12 months post (181) | | 36.4 (26.8 to 49.8) | 34.9 (27.4 to 45.6) | 0.59 |
| Post-implantation (350) | | 37.3 (23.5 to 51.5) | 38.5 (25.2 to 49.3) | 0.58 |
| *Ease of Communication (EC)* |  |  |  |  |
| Pre-implantation (255) | | 54.0 (30.5 to 74.8) | 54.1 (31.2 to 81.0) | 0.62 |
| 6 months post (239) | | 22.8 (12.0 to 37.5) | 25.8 (16.3 to 38.9) | 0.37 |
| 12 months post (181) | | 24.6 (14.2 to 29.3) | 18.5 (12.0 to 27.0) | 0.03* |
| Post-implantation (350) | | 20.7 (12.0 to 37.4) | 20.5 (12.3 to 33.2) | 0.96 |
| *Background Noise (BN)* |  |  |  |  |
| Pre-implantation (255) | | 74.5 (57.7 to 84.8) | 77.8 (64.3 to 91.0) | 0.05 |
| 6 months post (240) | | 54.0 (29.7 to 67.8) | 55.2 (41.5 to 72.2) | 0.07 |
| 12 months post (181) | | 49.8 (37.5 to 64.3) | 52.0 (37.5 to 62.3) | 0.77 |
| Post-implantation (350) | | 49.7 (29.0 to 64.5) | 54.2 (39.5 to 64.5) | 0.11 |
| *Reverberation (RV)* | |  |  |  |
| Pre-implantation (254) | | 76.7 (58.3 to 93.0) | 76.7 (61.2 to 93.0) | 0.71 |
| 6 months post (239) | | 50.0 (33.3 to 72.5) | 50.0 (37.5 to 71.3) | 0.78 |
| 12 months post (181) | | 50.8 (31.7 to 71.9) | 49.7 (39.3 to 62.5) | 0.50 |
| Post-implantation (348) | | 50.0 (28.5 to 70.7) | 46.8 (33.3 to 70.0) | 0.63 |
| *Aversiveness (AV)* | |  |  |  |
| Pre-implantation (255) | | 21.0 (10.2 to 42.6) | 29.4 (11.9 to 50.5) | 0.15 |
| 6 months post (239) | | 17.2 (8.7 to 35.2) | 22.8 (11.2 to 45.9) | <0.05* |
| 12 months post (181) | | 20.8 (8.8 to 35.5) | 22.8 (9.6 to 40.1) | 0.46 |
| Post-implantation (350) | | 18.7 (8.7 to 37.2) | 21.0 (7.6 to 39.5) | 0.54 |

**Table S2**. Distribution of characteristics and scores between tinnitus and no tinnitus reported post-implantation.

APHAB: Abbreviated Profile of Hearing Aid Benefit; CI: cochlear implantation; CNC: consonant nucleus consonant; IQR: interquartile range; n: number of patients; PTA: pure tone average.

* indicates variables that showed a significant difference between the groups (p<0.05)

| Tinnitus changes | Induction | No change | No tinnitus | Reduction | Suppression | Worsening |
| --- | --- | --- | --- | --- | --- | --- |
| Total ear (176), n (%) | 16 (9.1) | 49 (27.8) | 63 (35.8) | 15 (8.5) | 31 (17.6) | 2 (1.1) |
| APHAB, median (IQR)  Pre-implantation (163) | 72.0 (55.1-78.6) | 55.2 (45.0-69.6) | 60.7 (49.1-71.9) | 65.8 (53.0-69.3) | 54.1 (46.7-62.7) | 49.2 (48.6-49.9) |
| 6 months post (154) | 45.3 (36.7-57.9) | 36.0 (27.6-48.2) | 40.8 (25.9-51.1) | 49.2 (31.8-55.8) | 38.0 (23.3-52.6) | 53.3 (50.4-56.2) |
| 12 months post (118) | 44.1 (40.4-55.0) | 33.2 (26.3-45.2) | 34.5 (29.0-47.1) | 41.6 (31.0-49.8) | 36.4 (23.9-43.8) | 45.3 (45.3-45.3) |
| Post-implantation (176) | 39.4 (30.0-57.2) | 35.5 (24.7-45.1) | 36.9 (25.9-48.5) | 42.6 (31.5-59.5) | 34.9 (20.7-38.8) | 46.4 (45.9-47.0) |
| *Ease of communication (EC)*  Pre-implantation (163) | 72.7 (41.1-86.0) | 53.0 (30.5-77.4) | 57.2 (33.2-78.3) | 50.2 (33.0-72.8) | 52.1 (29.0-69.7) | 44.8 (38.2-51.5) |
| 6 months post (154) | 33.3 (20.6-38.5) | 22.8 (16.3-33.3) | 22.8 (12.8-39.0) | 27.0 (11.6-35.3) | 29.2 (11.2-41.7) | 41.2 (38.0-44.4) |
| 12 months post (118) | 22.4 (18.5-27.4) | 20.6 (12.3-30.3) | 22.7 (14.4-33.3) | 18.5 (12.3-29.0) | 18.5 (12.0-27.5) | 37.2 (37.2-37.2) |
| Post-implantation (176) | 17.6 (10.8-38.4) | 22.5 (14.2-33.2) | 18.7 (13.5-39.5) | 27.0 (14.2-36.5) | 16.3 (12.0-24.9) | 36.0 (35.4-36.6) |
| *Background Noise (BN)*  Pre-implantation (163) | 88.0 (71.1-93.5) | 74.7 (59.5-86.9) | 78.7 (66.2-86.4) | 87.0 (74.5-93.0) | 71.5 (58.2-80.8) | 80.8 (76.8-84.9) |
| 6 months post (154) | 64.5 (39.6-70.5) | 54.2 (40.0-64.5) | 56.2 (41.8-72.0) | 56.2 (42.7-75.7) | 45.7 (29.2-67.0) | 74.7 (67.5-81.8) |
| 12 months post (118) | 54.0 (45.8-70.5) | 51.0 (34.7-62.3) | 49.8 (37.5-65.3) | 60.2 (52.0-66.5) | 45.9 (31.8-59.3) | 66.5 (66.5-66.5) |
| Post-implantation () | 43.8 (38.4-64.5) | 54.2 (35.3-64.5) | 58.2 (38.5-66.3) | 57.8 (44.8-72.5) | 41.5 (23.0-56.2) | 63.4 (61.9-65.0) |
| Reverberation (RV)  Pre-implantation (163) | 87.8 (73.7-97.0) | 70.7 (55.7-89.5) | 79.7 (62.9-94.8) | 72.7 (57.9-90.0) | 74.7 (55.0-83.5) | 68.5 (67.5-69.5) |
| 6 months post (153) | 60.3 (45.2-68.5) | 50.0 (34.2-62.3) | 54.0 (37.5-74.5) | 47.8 (37.5-59.2) | 49.5 (35.5-84.6) | 81.8 (77.1-86.4) |
| 12 months post (118) | 50.0 (41.3-62.5) | 44.2 (37.5-60.9) | 54.2 (29.2-67.5) | 50.0 (48.0-54.8) | 47.6 (26.5-56.7) | 76.7 (76.7-76.7) |
| Post-implantation (176) | 47.7 (25.0-77.3) | 41.7 (33.3-66.5) | 50.0 (28.3-70.6) | 50.0 (33.4-67.4) | 41.7 (29.1-72.7) | 74.6 (73.5-75.6) |
| *Aversiveness (AV)*  Pre-implantation (163) | 26.1 (16.4-41.7) | 28.2 (13.1-38.1) | 16.8 (7.3-30.7) | 47.7 (25.9-66.3) | 32.4 (17.4-41.5) | 2.8 (1.9-3.8) |
| 6 months post (154) | 52.0 (23.8-70.2) | 14.2 (8.3-24.8) | 15.1 (8.1-31.3) | 45.5 (26.3-64.4) | 17.8 (11.2-41.8) | 14.6 (11-17.5) |
| 12 months post (118) | 47.8 (40.2-56.2) | 22.4 (10.1-28.3) | 14.5 (4.8-27.1) | 25.2 (14.2-35.2) | 26.9 (10.4-42.0) | 1.0 (1.0-1.0) |
| Post-implantation (176) | 41.5 (30.2-54.8) | 16.3 (8.3-26.8) | 14.2 (5.8-31.1) | 35.3 (18.7-57.1) | 12.3 (4.7-29.2) | 10.8 (5.9-15.6) |

**Table S3**. Distribution of APHAB total and subscales scores between tinnitus changes groups.
